# The experience of self-compassion training among NHS healthcare professionals

**DOI:** 10.1177/13591053241267041

**Published:** 2024-07-30

**Authors:** Sarah Wason, Ceri Sims

**Affiliations:** 1 Buckinghamshire New University, UK; 2 Buckinghamshire Healthcare NHS Trust, UK

**Keywords:** compassionate care, health professionals’ wellbeing, healthcare professionals, positive psychology, self-compassion, self-compassion training

## Abstract

Self-compassion in healthcare professionals (HCPs) is under-researched and undervalued. Promoting self-compassion within healthcare could have far-reaching benefits. This research study explores the experience of four NHS HCPs receiving a single short self-compassion training, with recommended at-home practices completed over 4 weeks. Subsequently, semi-structured interviews gathered information about their experience, resultant wellbeing and any impact on colleagues and patients. The main themes emerging from analysis of the interviews were motivation, permission and prioritisation. The motivation to practise self-compassion, and share this learning resulted from improved understanding of its potential benefits. Permission ties in with the notion of common humanity in self-compassion and its impact on negative self-talk and negative attitudes to self-compassion in a workplace. Prioritisation acknowledges the challenges for HCPs of investing time in self-compassion practice despite overstretched HCP workloads. Further consideration of these themes may help to better target any future research into strategies for enabling self-compassion among HCPs.

## Introduction

The discipline of positive psychology (PP), defined by Peterson (2006) as ‘the scientific study of … those things that make life most worth living’ (p. 4), offers an opportunity to understand and approach healthcare differently. It allows us to re-frame some of the major challenges facing medical systems around the world. Seeds of PP principles are already being sown by insightful commentators, one of whom recently went so far as to propose that loving one’s workforce was key to solving the global healthcare workforce crisis (Britnell, 2019).

Beyond the impact on the workforce, there is also an opportunity for PP to directly benefit patients by promoting emotional wellbeing, facilitating healthy lifestyles and creating physiological shifts which Lianov et al. (2020) suggest could address society’s top current lifestyle-related public health crises.

The evolution of PP into a second wave, embracing suffering and the whole person, and also a more recent third wave that includes a call for more exploration and focus on the interconnectedness of wellbeing among communities, means that the discipline acknowledges the often-severe hardship as well as socio-cultural complexities of people’s lives. Furthermore, the rippling effect of positivity (Fredrickson, 2013) enhanced by PP interventions, implies that if self-compassion is practised by HCPs, it could also indirectly benefit patients.

One positive emotion that features heavily in PP research is compassion (Gilbert, 2009; Kirby, 2017; Mascaro et al., 2020). Although compassion seems notoriously difficult to conclusively define, it is agreed to be a positive emotional response. Compassion is fundamental to professional standards in healthcare globally (Burnell and Agan, 2013; Ling et al., 2021). In fact, as compassion has received so much professional and scientific scrutiny and is felt to be fundamental to patients and HCPs alike, the evidence which indicates compassion to be somewhat lacking in healthcare (Ling et al., 2021; Lown et al., 2011; Papadopoulos and Ali, 2016) is particularly concerning.

Without underestimating the significance of organisational barriers to HCP’s compassion (Conversano et al., 2020), the study of self-compassion within PP has offered a new opportunity to better promote compassion. Studies have demonstrated that by cultivating self-compassion within HCPs, there may be an increase in the compassionate care that they can provide (Delaney, 2018; Mills et al., 2018; Neff and Pommier, 2013). This is in addition to the benefits for the staff themselves.

Research shows that the 2019–2021 Coronavirus pandemic has been particularly stressful for HCPs (Perego et al., 2023). Some argue that self-care has never been more important to enable HCPs to recharge and refuel (Bissett, 2022; Long Franco and Christie, 2021). Self-compassion sits among the tried and tested PP interventions which could offer much-needed support for the recovery of post-pandemic HCPs. However, PP interventions do not routinely feature in the current healthcare environment outside mental health clinical and research settings (Lianov et al., 2020). This is an area of huge potential and for which there is scope for further development. This study explores the experience of introducing HCPs to a small part of an existing evidenced-based self-compassion PP intervention, looking at the impact on HCP’s experiences of wellbeing, compassion towards others and patient care. The researchers hope that this will contribute towards improved healthcare services through more targeted use of evidence-based PP interventions.

### Self-compassion theory

Kristin Neff (Neff, 2003) spearheaded her work on self-compassion within positive psychology. Originating in Buddhist tradition, Neff proposed a new way of thinking about self-compassion in secular terms (Neff and Davidson, 2016). The definition is made up of three interacting components: self-kindness versus self-judgement; a sense of common humanity versus isolation; and mindfulness versus over-identification. Put simply, she sees self-compassion as ‘being touched by and open to one’s own suffering’ (Neff, 2003: 87). While this construct is not without potential limitations (Sinclair et al., 2017), there is now a large body of evidence linking the concept of self-compassion to wellbeing. Multiple studies have found that treating oneself compassionately when confronting personal suffering promotes positive mental health and also appears to promote resilience (Long Franco and Christie, 2021; Neff et al., 2020) by influencing people’s reactions to negative events (Smeets et al., 2014). Kirby’s 2017 article includes a useful overview of the impact of compassion-based interventions on brain activation and resultant well-being.

There is compelling evidence that self-compassion may be a prerequisite to compassion for others (Hofmeyer et al., 2016; Mills et al., 2015; Wiklund Gustin and Wagner, 2013). Neff and Germer (2013) demonstrated an impact of self-compassion training on feelings of compassion for others, hypothesising that this occurred due to ‘an increased perception of shared humanity’ (p. 40). However, one major limitation of these studies appears to be that no study has included patient reports to date (Sinclair et al., 2017).

### Self-compassion for HCPs

Currently there is an acute need for mental health support and self-compassion among HCPs. Even before the 2019–2021 Coronavirus pandemic, experts reported on the acute pressure faced by HCPs, which they attributed to perpetually increasing expectations of efficiency and production in the function of modern healthcare systems (National Academies of Sciences, Engineering, and Medicine et al., 2019). Such expectations are thought to have led to increasing workloads and the introduction of challenging technologies which negatively impact on patient care and personal and professional values (Neff et al., 2020). Andrews et al. (2020) go so far as to suggest that the pressure created by broken, business-orientated healthcare systems leave HCPs feeling like ‘automatons … with technical skills more highly valued compared to relational ones’ (p. 5). In combination with this increased pressure, a lack of supportive resources for HCPs is reportedly contributing to the accumulation of chronic work stress, poor mental health and burnout (National Academies of Sciences, Engineering, and Medicine et al., 2019). The pessimistic findings of research into HCP wellbeing are stark; for example, a 2018 meta-analytic review reported that worldwide, 34% of nursing students experienced depressive symptoms (Luo et al., 2019), while an earlier robust study reported rates of hospital nurses with anxiety, depression, burnout syndrome and posttraumatic stress as high as 87% (Mealer et al., 2009).

Within the limited research that does exist regarding self-compassion in HCPs, already clear links have been demonstrated between self-compassion and reduced compassion fatigue and burnout (Andrews et al., 2020; Bluth et al., 2021; Conversano et al., 2020; Delaney, 2018; Dev et al., 2020; Duarte et al., 2016; Long Franco and Christie, 2021). However, to date, even within the field of PP, studies with HCPs appear to focus more on prevention of pathology rather than positive promotion of wellbeing and the potential impact that this may have on patients (Delaney, 2018; Javanmard et al., 2021). This seems counterintuitive and may indicate the need for a change in focus to use self-care, including self-compassion, as a more ‘proactive, preventative measure’ (Andrews et al., 2020: 2) to have a wider positive impact. Nevertheless, research exploring how self-compassion and self-care influence the delivery of compassionate healthcare is limited (Andrews et al., 2020; Sinclair et al., 2017). This study begins to explore possible avenues for future research into this based on the insight gained from HCPs being introduced for the first time to self-compassion interventions.

Many challenges remain when it comes to attitudes within healthcare towards the idea of self-compassion. The scale of the challenge is demonstrated by a recent survey of nurses which found that 70% put their patients’ health, safety and wellbeing before their own (Bissett, 2022). A commonly voiced negative attitude is the notion that self-care is selfish despite the growing body of evidence suggesting that it is essential as a foundation for compassionate care (Mills et al., 2015). Another challenge is the sense that, particularly within nursing, the job role is a vocation ‘which brings with it an element of self-sacrifice’ (Andrews et al., 2020: 5), thereby impeding HCPs from accessing self-care and self-compassion. Cultural differences and previous self-care experiences have been shown to impact on the ways that self-compassion is experienced in HCPs, sometimes negatively (Bissett, 2022; Dev et al., 2020).

### Promotion of self-compassion in healthcare

Despite increasingly clear evidence of the positive effect of self-compassion, there is still surprisingly little focus within healthcare organisations on how to better promote self-compassion. Several effective programmes being developed within the positive psychology arena have proven successful in supporting the total wellbeing of HCPs and their patients (Lianov et al., 2020; Neff et al., 2020). Nevertheless, knowledge of how to cultivate self-compassion is generally still under-researched (Kotera et al., 2021; Rushforth et al., 2023) and research into self-compassion training for HCPs is particularly sparse (Neff et al., 2020). There is now evidence emerging from a recently developed Self Compassion for Healthcare Communities (SCHC) programme (Neff et al., 2020) around the effectiveness of shorter programmes of self-compassion training for HCPs (Long Franco et al., 2024; Neff et al., 2020). However, it is very much in its infancy. This study hopes to add further insight to inform this novel area of research, particularly within an NHS setting.

With the impetus currently within healthcare to introduce education and practice standards around self-care more generally, it raises questions about how effective this can possibly be without HCPs learning how to be self-compassionate (Mills et al., 2015). Proponents of self-compassion feel that it offers more than self-care alone and self-compassion practices are particularly advantageous because they can be used on the spot, potentially while at work, immediately when challenges are felt (Long Franco et al., 2024; Neff et al., 2020).

Self-compassion can be taught. Neff and Germer (2013) tested the effectiveness of their mindful self-compassion (MSC) training programme suggesting that MSC can promote various aspects of wellbeing including self-compassion, mindfulness and compassion for others and that this impact can last up to a year.

However, self-compassion training is, historically, long and time intensive. Neff and Germer’s (2013) gold standard MSC training requires participants to meet weekly for 2 or 2.5 hours over 8 weeks followed by a half-day meditation retreat. Such commitment takes up work time which could exacerbate existing stress for time-pressed HCPs (Neff et al., 2020).

Shorter programmes of self-compassion training and short periods of self-compassion practice have even been shown to be more accessible and feasible (Bluth et al., 2021) and increase self-compassion and produce sustainable mental health changes (Long Franco and Christie, 2021; Mantelou and Karakasidou, 2017; Neff et al., 2020; Shapira and Mongrain, 2010; Smeets et al., 2014). Even ‘brief exposure to the basic tenets of self-compassion holds promise for improving aspects of self-compassion’ (Toole and Craighead, 2016: 104).

The experience of self-compassion in everyday HCP practice, including cultivation of and barriers to its use are not well understood (Andrews et al., 2020). This study will aim to build on current research and improve the understanding of the experience of HCPs when it comes to self-compassion training delivered as a single short introductory training session derived from the MSC programme developed by Neff and Germer (2013). This has recently been acknowledged as an important process in contributing to hypothesis development when it comes to illustrating the effectiveness of Self-Compassion training (Long Franco et al., 2024).

In summary, the potential benefit of increased self-compassion within HCPs to themselves and to their patients could be far reaching and particularly necessary in the face of HCPs’ recovery from the COVID-19 pandemic. Self-compassion could also address the chronic lack of compassion within healthcare. There are not enough studies yet to explain how self-compassion could be best influenced within HCPs given the circumstances that they face when it comes to role expectations, resource pressures and technological advancement. This study addresses some research questions around the experience of receiving a 2-hour long training session about self-compassion and its impact on HCPs.

## Research questions

- In what way does learning about self-compassion impact on a HCP’s wellbeing?
- In what way does learning about self-compassion influence HCP’s feelings and behaviours towards colleagues at work?
- In what way does learning about self-compassion affect HCP’s experience of patient care?
- In what way do experiences of learning about self-compassion interventions influence HCP’s motivation to continue to use them and to share them with colleagues and patients?

## Research design

This qualitative research study explored HCP’s experience of learning about and practising self-compassion interventions. It included semi-structured interviews with four healthcare professionals who participated in a single 2-hour long self-compassion training session followed by an invitation to practise a set of simple on-the-spot self-compassion interventions as part of the session and then to continue to practise them regularly for a period of 4 weeks at home. The participants were interviewed about their experience of the training and their feelings about using and sharing the interventions as well as the impact of the training on their wellbeing and their relationships with others, particularly at work.

The researcher chose a qualitative method to gather rich data about participants’ feelings around their experience of learning about self-compassion. A semi-structured interview completed after the initial training session allowed for follow-up or additional questions to explore emerging ideas around the research questions above. Verbal responses were recorded and subsequently a hermeneutic phenomenological approach was used to analyse them following the process of Thematic Analysis, which offered a flexible and accessible way to identify, analyse and describe patterns, known as themes, within data (Braun and Clarke, 2006). Thorough records of the process were maintained using Microsoft Excel spreadsheets and along with handwritten mind maps, they provide an audit trail documenting the process of theme generation, including personal reflections. A sample of the datasets has been included as a Supplemental Material with this study.

### Participants

Snowball sampling was used to recruit participants for this study due to the difficulty accessing participants in the limited timeframe available with the ethical implications of completing research in the NHS. Initially, the researcher sent a gatekeeper letter and sample Facebook text to acquaintances, providing details of the study. These gatekeepers then invited HCP acquaintances to participate in the training and interview for the study. By using this snowball sampling method, the researcher identified the first four HCPs to respond to the invitation who met the required inclusion criteria, as detailed below. A pragmatic sample size of four participants was believed to allow the researchers to meet the main goal of this qualitative research, which was ‘to ensure that the sample size is small enough to manage the material and large enough to provide a new and richly textured understanding of experience’ (Sandelowski, 1995, cited by Fugard and Potts, 2015: 183).

The respondents who first emailed the researcher and completed the consent form participated as a homogenous sample by meeting the inclusion criterion of being registered HCPs, who had been working in their current role for a minimum of 6 months. They were excluded if they worked in the same team or department as the researcher. Four female registered HCPs, two nurses and two occupational therapists, who worked in a wide variety of physical healthcare settings in Scotland, Norfolk and London in acute and community NHS trusts participated. They worked mostly in patient facing roles although one manager with a limited patient-facing workload also participated. It was hoped that this variety in HCP roles allowed for a wide range of experiences and enhanced the transferability of the study findings. As it was difficult to arrange a single time slot to suit all participants, 2-hour training sessions were scheduled and took place for each of the four participants separately.

### Procedure

The 2-hour training session was based on Session One of Neff and Germer’s (2013) Mindful Self-compassion Programme, ‘Discovering Mindful Self-compassion’. It included four guided activities and opportunities for reflection, specific learning around self-compassion theory and discussion about home practice expectations.

The four activities are detailed below:

1. Brief meditation featuring an introduction to the three main elements of self-compassion.
2. ‘How would I treat a friend’ activity.
3. ‘Soothing Touch’ activity.
4. ‘Self-compassion break’ activity.

At the end of the training session, participants were invited to continue using the self-compassion interventions in their lives and at work for 4 weeks prior to the interview.

The first author, an HCP and qualified teacher of complementary therapy with experience of delivering meditation group sessions, provided the training.

The interview schedule, derived from the research questions, explored the experience of being taught about self-compassion and how that affected wellbeing, workplace relationships and patient care.

After the interviews were completed, thematic analysis (TA) of the interview transcripts was completed to identify recurring significant themes relating to the initial research questions. Using an essentialist/realist method, TA allowed for the reporting of ‘experiences, meanings and the reality of participants’ (Braun and Clarke, 2006: 81). A hermeneutic phenomenological approach was used to describe and interpret the participant’s lived experience (Dangal and Joshi, 2020). This semantic approach to TA allows for themes to be identified within the explicit meanings of the data, with no further analysis required (Braun and Clarke, 2006).

The study was approved by Buckingxhamshire New University Postgraduate Ethics Committee.

Participants gave their informed consent, and debriefing information with relevant contact information should participants feel they needed further support.

## Findings

Following the six-step process of thematic analysis, using coded spreadsheets, mind mapping and ongoing reflection, three main themes were identified in relation to HCPs’ experience of a single short self-compassion training. The main themes of Motivation, Permission and Prioritisation were identified. They were noted to be factors impacting on the participants’ experience both positively and negatively and they were not time dependent. Table 1 below gives an overview of these themes and they are discussed further below.

**Table 1.** Key themes in self-compassion training.

Experience of self-compassion training	Motivation	Value of self-compassion practice felt so desire to learn more and share learning with others
		To practice self-compassion more
		To be compassionate to others
	Prioritisation	Impact of work environment and other conflicting priorities when it comes to finding time for practice
		More time given to practice then more benefit felt
	Permission	Allowing self to see self-compassion differently
		Allowing self to feel and respond to negative feelings differently

### Motivation

#### Motivated by learning the value of self-compassion

Participants described being motivated by an improved understanding of the value of self-compassion provided by the training session.

I suppose … it’s given me … a bit more of an understanding of what it means and why we need it … and … it sort of creates a bit more weight to it, which is really helpful … . (Participant 3)

An understanding of the wider application of self-compassion motivated participants to consider using the interventions in a variety of settings.

I’d maybe thought about it [using self-compassion] more … if I was not liking the way I look … I hadn’t thought about it in the way that I was annoyed with myself about work or felt I hadn’t done something very well … . (Participant 2)

These new ways of thinking about the value and application of self-compassion seemed particularly important for combatting commonly held views that self-compassion is selfish or self-indulgent.

it’s trying to remind myself that, … it’s not self-indulgent, it makes us better, to be around … . (Participant 2)

Mills et al. (2015) stressed that HCPs needed to change their view of self-care as selfish before they would be able to achieve the necessary balance in caring required to provide ongoing compassionate care. The training seemed to influence this change of self-care mindset in the participants.

#### Motivated by feeling the benefit

All four participants described feeling immediate benefits while practising self-compassion interventions in the training and soon after the session. Many of these benefits related to their hedonic wellbeing, focusing on happiness and defined ‘in terms of pleasure attainment and pain avoidance’ (Andrews et al., 2020: 141). Examples of these benefits are shown below:

I was pleasantly surprised about how certain activities made me feel. (Participant 1)

Self-compassion is quite specific around some of the physical feedback that you can have … I feel soothed definitely by doing that … . And calmer, calmer maybe. (Participant 4)

Feeling such positive emotions seemed to build the motivation of the participants to continue to practise self-compassion interventions, providing longer term benefits. Frederickson’s Broaden and Build theory of positive emotion which argues that positive emotions broaden our mindset and build personal resources over time (Fredrickson, 1998), provides a theoretical framework to explain this finding.

Other longer-term benefits of the self-compassion training included increased self-awareness:

It was a really nice, good positive experience having an allocated time to think about the impact of how you view things yourself. (Participant 4)

Participants variously described how this awareness prevented their spiralling into a negative dialogue and feeling less self-critical, more confident, more self-reliant and more compassionate.

Because I’m feeling calmer … when I move on to the next patient. … I don’t know if you could quantify that, but I wonder if it has made me calmer with that patient cos, if I’m a bit rattled … and then I did your technique and just took a moment before I then spoke to the [patient with the] sore throat. I wonder if … I’m kinder. I’m a bit better. I’m not as rattled. (Participant 2)

To be honest, probably it has helped me become more assertive. (Participant 1)

The apparent increase in participants’ self-awareness achieved after this short self-compassion training session is encouraging and could potentially also impact on patients. Andrews et al. (2020) argue that ‘if nurses are able to manage the emotions of caring by being self-aware and recognising when they need to apply self-care and self-compassion, then they may feel more able to offer compassionate care to others’ (p. 2). Other studies have indicated similar positive change in HCP’s attitude towards patients following short self-compassion interventions (Bluth et al., 2021).

#### Motivated to be compassionate

Participants clearly described a relationship between self-compassion and the delivery of compassionate care (Mills et al., 2015), with its multiple advantages for both patients, colleagues and HCPs themselves:

Knowing about the importance of self-care made me think, … . it’s important too for my patients … Your talk and my knowledge of things did probably make me feel … when you have patients that are elderly or whatever … you think, hang on a minute, … let’s look after them. (Participant 1)

You’re just much more open to nurturing, when you feel looked after and nurtured and do self-care. (Participant 2)

#### Barriers and challenges

Motivation to continue to practise interventions may have been improved if written content was provided before and after the session, to support the training.

The main barrier was completely forgetting to do it … It might be useful to have had a, like a written prompt or reminders to support training.

### Permission

The self-compassion training seemed to offer participants the permission to think about their own self-compassion, to look after themselves and to self-soothe.

It was a really nice, good positive experience having an allocated time to think about … or validate the fact that it’s OK to have time to support yourself. (Participant 4)

Several participants felt that before the training they had never reached a point of being able to give themselves permission:

I just never had given myself permission … to allow myself some self-care … I just think I’ve been through some things, and I just think, nothing can make me feel better … you helped me realise that actually, you can give yourself a hug. You can, you can do that and that’s fine. (Participant 1)

Andrews et al.’s (2020) study of the experience of self-compassion in nursing similarly found that permission is required by nurses to be self-compassionate. They also noted that even having permission to talk about self-compassion can be ‘cathartic’ and ‘caused them to think about how to care for themselves more in future’ (Andrews et al., 2020: 3). The process of training about self-compassion offered participants this opportunity.

#### Permission to feel negatively

The experience of self-compassion training and practice seemed to give participants permission to rise to the challenge of sitting with negative feelings.

So, it’s like understanding why you feel a certain way … . And knowing that you’re allowed to feel a certain way, there’s nothing wrong with it. Everyone feels that certain way. (Participant 2)

This newfound permission seems related to the reminder, through self-compassion practice, that you are not alone in whatever negative situation you may be facing. This is in line with Neff’s (2003) ideas of shared humanity as a core part of self-compassion. Appreciation of this shared humanity then enhances our sense of connectedness with others potentially increasing our compassion towards others (Neff and Germer, 2013).

#### Permission to change

The challenge of giving oneself permission to become more self-compassionate was acknowledged by participants, particularly at times when self-awareness may be lacking. This could affect how people approach the training and how much practice they may feel able to do. Participant 3 described how:

It’s about being kind when you’re not feeling right and if you’re not able to acknowledge that you’re not feeling right then, or you’ve got real blocks around it, that would be quite hard, I think.

Tackling long-standing, deeply entrenched negative feelings about self-compassion or negative self-talk can be something that requires a lot of practice, as acknowledged by Participant 3:

Because I am quite self-critical, therefore … there is a value for me to have that self-compassion practice every day … . it does need time investing in it … it wasn’t something that was natural to me and not something I was brought up doing and therefore, … it has taken some very definite practice.

Important issues concerning participants’ readiness to give themselves permission to be self-compassionate were raised. These require further exploration, particularly given the wide-ranging benefits that participants may gain from allowing themselves this opportunity.

#### Permission from others

Permission to be self-compassionate at work was identified as important.

Being fair to myself … maybe not as harsh on myself about what needs to be achieved or what can be achieved … and being allowed to think about that being important within the working day… that would be positive.

Role-modelling self-compassion could be one way to demonstrate this permission to others. Mills et al. (2015), understood that nurses could exemplify health promotion by practising self-care themselves. Participant 4 felt that the self-compassion training allowed them to better understand the value of self-care practised by other HCPs.

it gives me a greater understanding of other things that people might be doing for themselves to look after themselves. (Participant 4)

The value in self-compassion training can therefore potentially spread beyond the individual and contribute to a wider culture change.

Situating Self-Compassion training programmes within healthcare settings may help to enhance their effects (Long Franco et al., 2024). Andrews et al. (2020) concluded that ‘if self-care and self-compassion were more understood and embedded within nursing education and the workplace culture, this would serve as a form of permission’ (p. 7). Long Franco et al. (2024) suggest that ‘investing in a culture that exhibits and encourages compassion to oneself and others’ (p. 366) shows support for HCPs emotionally. Development and promotion of a short self-compassion training programme, such as the one completed for this study, could facilitate the process of embedding self-compassion within the workplace culture, thereby permitting HCPs to be self-compassionate.

#### Barriers and challenges

The workplace culture or attitude of colleagues could be one barrier to feeling able to practise self-compassion at work. Participant 2 identified that colleagues may not be ‘*safe*’ to speak to about self-compassion at work.

I would be cautious … who I talked about it with because it is very easy to be mocked, … I can think of two or three people right now who … would be annoyed if I started talking about it.

It is notable that the lack of a support system or a positive relationship with co-workers has also been found to reduce compassion satisfaction (Hinderer et al., 2014), which is linked to reduced self-compassion (Neff et al., 2020). This highlights the importance of tackling any negative culture of HCPs towards self-compassion.

### Prioritisation

The prioritisation of self-compassion practice, particularly that of regular practice was another theme to emerge. After better understanding the value of self-compassion after the teaching, participants noted how they had started to prioritise regular practice of self-compassion:

[I] spoke to one of the senior nurses because the teaching has been really effective, and it just brings home the importance to me of making sure that you do fit it in your life when you can. (Participant 1)

Prioritising time for self-compassion initially seemed to help practices to develop:

It got better as time went on; it became easier to do … . As the more you did it, the more instantly just that process would make you feel a little bit calmer within yourself … So, it was like I knew what was coming. (Participant 2)

This experience was likened to HCP’s annual cardiopulmonary resuscitation training, which requires an initial investment and prioritisation of time to learn the basics and then only short top-up sessions are required to rekindle the learning and feel the benefits.

#### Impact of circumstances

Awareness of how circumstances affected their ability to prioritise self-compassion practice was shared by all participants. Neff et al. (2020) acknowledge the ‘intense demands of the job (of HCP)’ (p. 2). Participants described ‘busyness’ at work, caused by the pressure of the workload, which at times felt overwhelming. Participant 1 described the competing priorities of work and practising self-compassion clearly as follows:

Well, time obviously is a big barrier … when you’re working because you’ve got 50,000 other things to think of … So, it’s really difficult to step away from that. You have to force yourself, basically, I think, sometimes.

Competing priorities also result from demands at home:

there’s new priorities that, that come up in work or personal life (Participant 4)

These competing priorities resulted in participants feeling unable to remember the benefits of interventions, feeling unable to focus or to calm their mind and feeling that they were not practising self-compassion properly:

… like it’s a really busy day. And everything’s all firing about my mind trying to settle down, to do it, I find like I’m not doing it properly … I can’t. Everything is too busy. So sometimes that got in the way … (Participant 2)

Because of the nature of the role, Participant 2 felt the need to be available at work, at all times, so could not take any time out for self-compassion practice:

The doctors can call you whenever and stress … practising poor time management by taking, sort of, selfish time for myself, although it’s essential, that … could then stress me out at a later point.

Attitudes like this could be part of the reason for the risk of burnout in HCPs being noted to be so high (Neff et al., 2020). By the same token, such stressful circumstances at work were noted to make compassion and self-compassion more important. This raises questions about how to facilitate prioritisation of self-compassion.

#### Enabling self-compassion practice

This study, along with others, seems to offer hope of the potential for self-compassion skills to be learned ‘in a format that is more conducive to the time constraints of a healthcare setting’ (Neff et al., 2020: 14).

To have something that is accessible and short and manageable and has a power to it: you notice a difference. I think that’s such a lovely entry point. And I think for me. That’s how I started. (Participant 3)

Participants offered several ideas of other strategies that help them to prioritise self-compassion practice, which would be useful to note if planning any future training. These included having an allocated place to practise (Participant 3). Having reminders in place to practise regularly, including reminders that practice is ‘non-negotiable’ (Participant 2).

Incorporation of self-compassion into other previously learned meditation or self-care practices was noted to be helpful and meant that participants had already found time and made a habit of regular practice:

Like the breathing I was already doing with the meditation, but having the touch there as well, like a trigger and … . I don’t know why it felt so relaxing, but it was. (Participant 2)

Several participants suggested incorporating self-compassion practices into some sort of routine to encourage more regular practice:

a lot of it is quite entrenched in habit … .so I think that is an enabler. Is routine for me … . Routine, regularity. And I think if, without that I would be much more rubbish at it. (Participant 3)

Other researchers have understood the value of routine for regular self-compassion practice and Bissett (2022) provides other useful suggestions for how to make self-compassion more routine. Shift-work and flexible working patterns experienced by HCPs mean that a lack of regular working times and days makes routines difficult, and Participant 4 noted this to be a barrier to regular self-compassion practice.

## Discussion

This study allows us to begin to build a picture of the challenges and opportunities for HCPs in the UK when it comes to experiencing self-compassion. It contributes to the limited knowledge that exists about the experience of self-compassion in HCPs, including the way in which it can be cultivated and the barriers to its use in everyday healthcare. The dearth of knowledge in the healthcare domain has been acknowledged (Andrews et al., 2020; Long Franco et al., 2024; Neff et al., 2020; Prudenzi et al., 2022) with the coronavirus pandemic compounding an already difficult picture of mental wellbeing in HCPs. The current research adds to the growing evidence pointing to the potential for HCPs’ self-compassion to impact on their own and their patient’s wellbeing (Long Franco et al., 2024; Raab, 2014).

In this study, participants had limited knowledge or understanding of self-compassion before the training session and so came to the experience with few expectations. They unanimously described having their eyes opened to its potential benefits. Participants found the experience to be positive and were unable to identify any negative feelings related to the interventions practised.

Overall, the multiple beneficial effects described by participants included some with immediate positive feedback, or hedonically pleasurable experiences. Participants also identified longer-term benefits from regular self-compassion practice, which appeared to be associated with increased self-awareness and self-reliance, resulting in less self-criticism, less negativity and increased self-confidence. They also provided some notable examples of when these benefits could be shared among people in their daily lives, both at work and home. Mention of increased compassionate feelings towards others seemed particularly significant for HCPs since it perhaps demonstrated how training could impact on compassionate patient care.

This research brought to light key themes influencing the experience and whether the continued benefit of Self-Compassion practice was felt beyond the initial training. These were Motivation, Prioritisation and Permission. Motivation relates to motivation to practice, to learn and to share the learning. Permission related to being allowed to see self-compassion differently and being allowed to feel and respond differently to negative feelings. Prioritisation relates to investing the necessary time in the practice to get the most out of it, finding time to fit in it. Other factors which appeared to affect the experience for participants included the personality of the participant and their circumstances, including the people around them.

The brief teaching of self-compassion did appear to be of benefit both immediately to HCPs and potentially in the long term. By exploring the effect of self-compassion on HCPs in greater detail, taking into account issues around motivation, permission and prioritisation may help to target future training programmes optimally (Prudenzi et al., 2022).

When it comes to adopting a more self-compassionate approach to life and work, HCPs are faced with unique challenges concerning their own and society’s expectation of themselves along with the potential for enhanced benefits beyond the individual. Some challenges appear to directly impact HCPs’ ability to implement self-compassion into their working day. This is unfortunate when it may be of most benefit to patients if used at these times. This study begins to explore how the introduction of short training programmes could help to address some of the challenges faced and potentially this could bring about an increase in the compassionate care of patients.

### Implications for future research and practice

It is important to note that several factors may have influenced the results above. Because the researcher conducting the training and interviews (SW) was an HCP, it is possible that interpretation of the data was affected by their own preconceived ideas of working in the field, although their immersion in healthcare in the NHS for many years may also have been beneficial in terms of providing some insight into the setting. The support of a university supervisor (CS) who was not an HCP may have helped to reduce any such bias. In future, research bias could further be avoided by involving an independent researcher for interviews or data analysis and member checking. There is a risk that participants’ responses were influenced by their relationship with the researcher and their desire to ‘say the right thing’.

Although the sample was small, the participants included a disparate set of roles and positions within the NHS. While these research findings cannot be generalised from such a small data set, this study has sought to capture deep and rich data to inform directions for future much-needed research. Generalisability could be improved in future by including a wider participant pool, including more of an ethnic and gender mix. The authors would recommend that resulting themes and insights be more robustly tested using a mixed-methods approach, perhaps by approaching nominated teams of NHS patient-facing HCPs to answer specific questions about the impact of single short self-compassion training programmes on wellbeing and compassion towards others, with the addition of measures of any change in compassionate care outcomes taken from patients.

Useful suggestions were made about how to improve the training session. Provision of written reference material may have enhanced the participants’ learning. Encouragement to structure self-compassion practice, for example by fitting it into a routine already in place or setting digital reminders, may have helped the participants to practise more consistently, leading to better outcomes.

Nevertheless, this study adds to existing evidence that increased self-compassion and compassionate care can be achieved even with a small amount of training of HCPs. There is the potential for a much wider reach when it comes to learning about self-compassion, its benefits and techniques for enhancement among health care providers worldwide. Better understanding of what motivates HCPs to learn about self-compassion and how they can feel permitted to practise even in situations when they need to make difficult decisions about prioritising self-compassion over other important work responsibilities could be the key to creating a more compassionate culture within healthcare.

## Supplemental Material

sj-docx-1-hpq-10.1177_13591053241267041 – Supplemental material for The experience of self-compassion training among NHS healthcare professionals Supplemental material, sj-docx-1-hpq-10.1177_13591053241267041 for The experience of self-compassion training among NHS healthcare professionals by Sarah Wason and Ceri Sims in Journal of Health Psychology

sj-xlsx-2-hpq-10.1177_13591053241267041 – Supplemental material for The experience of self-compassion training among NHS healthcare professionals Supplemental material, sj-xlsx-2-hpq-10.1177_13591053241267041 for The experience of self-compassion training among NHS healthcare professionals by Sarah Wason and Ceri Sims in Journal of Health Psychology
