# Supplementary material for: The experience of self-compassion training among NHS healthcare professionals
Source: J Health Psychol. 2024 Jul 30;30(6):1227–41. doi: 10.1177/13591053241267041 (PMC12052951; doi:10.1177/13591053241267041)
Supplement: sj-docx-1-hpq-10.1177_13591053241267041 – Supplemental material for The experience of self-compassion training among NHS healthcare professionals [file sj-docx-1-hpq-10.1177_13591053241267041.docx]

I
Come up on your screen. And then it will, it does all hard work for me. You see, so that's brilliant.

R

I didn’t know and I think that's great.

I
I know, it's great. I um, I've used it once before and I was like, is it gonna work? Is it gonna work? And then obviously, um it it it wasn't working when I first logged in and I thought, here we go, but actually it does seem to have worked on there, doesn't it?

R
Perfect.

I

So are you OK to carry on and do these questions for me? That's very important.

R

Absolutely. I'm fine. Yeah, good.

I

It shouldn't. It shouldn't take too long. As I say, about my, hopefully less than an hour, but um, I I've just got to, Umm, let's go to my official document here and see what it says. It just says um, so as a reminder, this interview is about your experience of participating in a training program of self compassion interventions and lasts less, just around an hour max. I just want to check you have done the consent form, didn't you? And that you're aware that you're not obliged to answer any question you don't want to and you can stop the interview or pause for a break at any time without giving a reason. That's fine.

R
Yeah. Yeah.

I

And so, yeah, the video audio recordings will take place during the interview in order to come back, for me um to come back and analyze any emerging themes is what I'm looking for. And it will be anonymized. So that's also good to know. And so yeah, hopefully you'll feel as comfortable as possible just to speak freely. So. So I've gotta ask, how did you, tell me about your experience of learning about self compassion? How was it for you?

R
I absolutely, really loved it. I've had other things going on lately with with work and things, just about my hours and things. I didn't feel so much compassion until I feel, explained, and I don't think I should always, we shouldn't always have to explain ourselves, sometimes but I think we should just hopefully have a little bit of compassion, but it obviously it doesn't always happen.

I

Umm.

R

So you you're teaching was perfect.

I
Brilliant, did it, did it kind of meet your? Did you experience uh meet or not meet, maybe, your expectations of the session? Did you have any expectations, do you think?

R
Do you know what, I've been thinking about things and I and anyway, I truly didn't have any expectations because I know this sounds awful, but I'm a little bit cynical. I just think I've I've been through some things and I just think, nothing can make me feel better. I just have to be hard on myself. I don't know why I think that, but I do. And. And you helped me realize that actually, you can give yourself a hug. You can, you can do that and that's fine.

I

That's good to hear. And did you come across any kind of barriers or challenges to, in, in the the session or after?

R

Not so much in the session, but I do think, obviously there are some challenges putting that into practice in the real world. Is that what you're looking for or?

I

Yeah, yeah, exactly. Just your experience about, of learning about self compassion, going back to that, what were the kind of challenges or barriers?

R

Yeah, um, only the, well time obviously is a big barrier and you’re just not, you can't be selfless, you just can't when you're working because you've got 50,000 other things to think of. Different patients, to do, on different wards and things, you've gotta go and see. And there's always something, or that follow up things that you think: Ohh yes, I forgot to put that in the notes or I must go and do that. And so these things are going on in your head. So it's really difficult to step away from that. You have to force yourself, basically, I think, sometimes.

I
That's good, yeah. So that that's really interesting. It's really, is it everything you're saying is really interesting so thank you, it's it, it can you describe any positive experiences? I think you maybe already have a little bit just at the beginning when you talked about work. The, but can you describe any other, any positive experiences with the learning and? Any others?

R

When you went through with me, sort of, certain, when we had the discussion last time and you went through with me hugging yourself and then. Just uh, all the different actions and things. Um and, sort of, I don't know, just kind of like, whether you close your eyes or open your eyes and different things. Um, like I've really enjoyed hugging myself, but not just hugging myself, I think, putting my hands right round myself and um, sometimes just, just, you, people don't understand what you're going through. So for you to give yourself the acceptance to do that, you are allowing yourself. So if you can reach, reach the point that you can allow yourself. Then I think you can, most of the activities you, were helpful, but some more so than others. Does that make sense?

I

Yeah, yeah, yeah. You were describing about your negotiations, I think, with work weren’t you, about your hours and how, you know, is, do you think that that, would you describe that as a positive experience? I don't know how, you were talking about relaying compassion into that kind of conversation and how that what, how that kind of played out for you, I don't know whether that was because you felt that you were able to be more compassionate with yourself or just.

R

I didn't think that, I didn't felt understood. I really didn't felt understood and I thought, but you have to understand things. I'm covered by the discriminate learning and by the Disability Discrimination Act just because of my head injury. I don't know if you need that or what, but, so I wanted to relay to them: you have to understand this, I'm not saying this. I'm not selfish at work. I'm saying this because I want the best for my patient. For, for me, myself and for everything. And I'm saying this to be safe and I need you, I need you to listen to me. And I found myself repeating the same conversation. And actually sister called me into the office and said Ohh, you've mentioned it to different people and I was like, I said to her sorry, um, yes, because there's been so much water under the bridge with with lots of situations at work, um that it caused an anxiety and you cannot, you cannot account for anxiety. And we're only human beings. We do do funny things sometimes. We're not superhuman. And the allowance for that, I I don't. I don't think there was an allowance for that really. Did that help? Does that make sense?

I
Yes, it sounds like you've become, like you've just sort of, almost. It. There was an assertiveness there that maybe, you felt that it helped you to keep pushing for what you needed, right? You were, to give yourself that compassion, that time to make sure it happened for you. That's great.

R

Yeah, yeah. I I really feel if you're not assertive in our profession, with yourself. You will get pushed to the curb. Just, I don't know if that even makes sense, but I think you can't be, because it's important.

I

Umm. I agree, yeah, I'm, I'm with you. So just the last question about the kind of learning and the experience of that learning. Was there anything that surprised you? Anything, like, that would stand out as a kind of, oh, that was really unexpected?

R

In a positive way or negative way?

I

Either either.

R
I was I I, honestly [researcher] I was politely, not politely, I was pleasantly surprised about how certain activities made me feel and talking to myself as if I was somebody that I was caring for or a friend. And and thinking hang on, you can talk to yourself in that way, that, I can't deny that did pleasantly surprise me um that I could, I could do that. It sounds ridiculous, but I just never had, never had given myself permission enough to allow myself some self care. Does that?

I
Thank you. Yeah, that's great. Umm, so did you. How did you feel about, uh, the regular self compassion intervention home practice? Sort of, how did, so in terms of home practice, did you, did you feel that more positive, you said already you felt more positive about the hugging and the and and, do you know why that, why you feel more kind of positive about that one or were there any others that you felt were more positive than?

R

Yeah, I I thought, um, I I also was pleasantly surprised about the giving myself the, you know, the the the words that I would give to another individual or friend, um, I was pleasantly surprised about that as well. The two, I would, there there wasn't one that really stuck out more out of those two things. Um, just knowing that you had permission to talk to yourself like like anybody else. It just didn't even occur to me, I guess maybe.

I
Is it is it? Is there a reason, kind of, why you think you felt more, those were more easy to put into practice, I suppose, at home or?

R

Definitely the hugging was when I put my hands right round myself. It really felt all engrossing. All, completely. Um, almost like I was, I I wasn't me. I was, I was somebody else hugging, because I wouldn't normally put my hands right round the back of myself unless it was my husband or something. And so, I don't mean that to sound weird but.

I

I think it's fine.

R

But but it did make me feel, um, it did, it did make me think, hang on, I am being cared for. And it's it's strange. I can't, I can't articulate why.

I
No, that's OK, that's fine. And did you, did, is there anything of the practices that you felt, kind of, more negatively about and can you, kind o,f is there any way you can describe why you might have felt that they were not so easy to put into practice at home or not the ones, you know, is it, did you have any, was there any reason why some of them you kind of left behind, do you think?

R
The the ones that weren't engrossing and all and, sorry, all engrossing and all, sort of, all encompassing, if you like. Um, I didn't worry about putting them into practice just simply because I didn't find them effective.

I

That's fine. Yep.

R

Um and I I just, it it's almost like, well, if they're not effective when, then why, why why do I need to? Why, how would that help me?

I

Yeah, that's good. Yeah, no, that's really helpful. And um did you, at home, with any regular practice that you did, did you, I'm just trying to work out what challenges there were, if any, for you in terms of, I think you've talked about that already, in terms of time and then, you know, just um, sort of more kind of regular practicing, did you, did you manage to? Was there any challenges or benefits that you got, that ohh over time, I suppose, as time went by more, the more you did it, did you notice a change or less less, doing it less frequently as you went as time went by or?

R

I I did things less frequently as time went by, but I didn't need to do it as frequently, um, because,um, it it was OK. And generally my whole persona and and being aware, I think self awareness about the whole thing, that you can do that whenever you need, you don't, you don't need to, you don't, you don't need to necessarily schedule a specific time because you can, you can do it whenever. Saying that, it makes me think, that's really, that's fine for me because although time is an issue, that's fine because I haven't got any children here and I can go upstairs. And shut the door. And, I don't know, sit in my chair or wherever it may be, when I, when I'm at home, when I want to. Um, whereas if I had children or other things here and noise, for example, then I don't think I would be able to so that, I don't know if that helped me out a little bit. It probably did. I felt it did.

I

OK, that sounds great. So just next, to thinking about your feelings about self compassion and it, how that changed? It, just about the, sort of, subject of self compassion and what it means now? Did you get, sort of, what were your main take home messages, if you like, from the training session, that you, is there anything that you haven't you think that you need to, I'm not sure if you've covered those already? But, do you know what I'm trying to, uh, do you, kind of, get what I'm trying to get at? Is it, what, in terms of the learning specifically, the messages that you got, how did you feel about those?

R
I think, I think I get what you mean. I. It was interesting, really interesting at first, but then ohh yes, that was what I was thinking. Um, I I thought I could just practice that whenever. But, sorry, going back a tiny bit, I couldn't practice it whenever, when actually there's been some other major issues going on in my head as of late. And I need to be in the right place.

I

Yeah, yeah.

R

Because I didn't even, I would be like, O, that, don’t, don't be silly, I haven't got time for that. That doesn't help me right now. That doesn't help me enough. But um, so so right place. But generally, if I don't have anything, I mean most things, OK, but if then, anything in particular on my mind then it's very difficult to focus in. But generally, because things weren't so bad generally, I could I could practice self compassion. Does that help?

I
Funny, isn't it? Yeah. Yeah, yeah. I I'm totally with you on that when you need it most, almost you don't, ou know, it's most difficult to do.

R
Yeah, yeah.

I

That's what, that's what I find strange, but, um yeah, that's good, in in terms of the subject, when you start, like imagine before, when I said, when you were approached by [name of acquaintance] and talked about self compassion and thinking about what you thought about self compassion then and then compared it to what you feel about it now, do you, do you have, do you feel like that's changed or your understanding has changed or your?

R

Yeah, pleasantly. Um, I've had a student nurse, actually as of late. And actually, one of her learning outcomes was to practice self compassion or to, not necessarily to practice self compassion, but is she? Is she able to? You know, to to to be compassionate to herself. Well, yes, self compassion to when when emotional events occur that really, really was one of her questions.

I
Wow.

R

I know. And I was like ohh my goodness, I know this. That's great. And I even spoke to one of the senior nurses because the teaching has been really effective and it just brings home the importance to me of making sure that you do fit it in, fit it in your your life when you can.

I

Yeah, that's good. That's it. And so like as compared to self care generally, do you, do you, are you, would you be able to, kind of, define how self compassion fits in that, in the subject of self care or how it's different from how it's more specific, are you able to define it? Would you be able to, if somebody at work or, you know, your, would you be able to kind of say well self compassion is this whereas self care is that or have it, do you think you were left with that kind of level of understanding or would you, is it something that you think you might need to? I'm just trying to get a sense of.

R

Yeah.

I

And what you are left with in terms of understanding I suppose?

R

I would think self compassion is about allowing yourself and giving yourself permission. And self care is the action, to me, of of looking after yourself. To me, I think. I don't know.

I

OK, good. No, it's not, it's not test, it's not. It's just, it's just, it's just it's interesting, it will, it will be, it will be helpful just to kind of understand how, that, you know, that that. It's difficult to to. It's a really difficult thing to define, I find, and it's just understanding how that, like one little bit of information that I've given you, a tiny bit, sort of settles, if you like. So it's not a test, it's just to see where that, you know, whether that where, where that feels a few weeks down the line, you know. So that's all good.

R

I I I yeah, I do think it's self compassion is the action of it in my mind and self care is doing it. I think, I think.

I
OK. How do you? So there's quite a lot of repetition here, I'm afraid, but it does. There's like, how do you feel about what you learnt about the intervention specifically? And so what do you, do you think of the self compassion interventions that you tried, we talked about that a little bit already? So I think we’ll, that unless there's anything else you wanna say about the ones that you tried, I think we talked about that, haven't we?

R
Yeah. Um yeah, I I, I I did just think that the hug, they they were nice, but the hug right round, UM me was good and talking to myself.

I

OK.

R
Yeah, it, that they were the most effective and I think sometimes, um just just like stroking, stroking my head. Umm. I do that a lot actually. And I and I sometimes find that quite helpful actually, thinking about it now.

I

It's funny, isn't it? Because then you you the. Whereas before you would have done things already that you just, sort of, do naturally and always have done, I think what’s quite nice after a while, for me anyway, was to go. Oh no, that is that's a little bit self compassion there I've just given myself that's, you know, and it kind of feels, you know, that that it almost makes it feel doubly meaningful if you like.

R

Yeah.

I

And I think that's.

R

Reminds you what you're doing and it justifies your actions, I think, yeah.

I

Yeah. Yeah, exactly. Umm, so we talked about, I think, the next one we've covered the ones you're more inclined to practice at home. Those are the the hugs and how do you feel that they changed you any of these interventions changed you at all, if at all I mean?

R

Um. So that's a bit difficult. Yes, in as much as I do think that it it reminds me to um to give yourself the time and how important it is. So I mean I I did know that, but I I can't say that it was articulated to me or it was gone through with me, or I really realised the importance. I just thought it was good and nice, but I think it just brought home the real importance, I think. And the ease, the ease that you can do it and it wasn't too difficult to me personally. Just fine throughout but Yeah.

I

Yeah, that's good to hear that it wasn't difficult and it's also, you know, I think we talk, what, you know, that the, I think the example that, again I'm gonna go back to the, I know I'm, the one that you gave at the beginning, maybe, about feeling a bit more assertive and being able to give yourself that time, that permission to do to to to to make sure that you're you're allowing yourself that self compassion.

R
Yeah.

I

You know, wherever you can, in whatever situation you can and. So yeah, hopefully that might. That might also be an example. Anything negative that you noticed? Anything that changes, like?

R
Not. Not really. I just, if it wasn't particularly helpful anything, any part of it, I just wouldn't do it. Um there, I guess the only negativity would be the the the time and the difficulty if you've got other things on your mind. If you've got significant other things on your mind. That would be a negative trait, a a negative part of being able to undertake the action, but I didn't experience any difficult any any difficulty or any, um any negativity, I guess, towards doing self compassion.

I

That's good because that, you know, because obviously at the beginning I think I said, you know, you could end up upset or, you know, there could, there there were situations which might have triggered things you know. So those are the, that's the issues that, I was suppose. As long as that isn't some, I, you know, it's nice to know that that hasn't become something that's put you off or.

R

To be honest, [researcher]. I really am one for being cathartic. And UM, allowing yourself, because if you don't, it will fester. It really will. And so you have to, you have to just embrace it. Um, and I think I think it has taken me years to arrive at that, um at that thought. But yeah, that is what I feel. I think.

I

That's good. It did. It. Did you find an opportunity or find, have a feeling about introducing self compassion into your working day, specifically, more like how it impacted you at work, if at all. Were you able to introduce any interventions at work? You're, for yourself.

R

The, to be honest, probably it has helped me make make, helped me become more assertive. I I can't say definitely that is what made me more assertive at work or that, um or that other things made me feel more assertive at work because there have been some other things that may have helped, but definitely, it definitely contributed to allowing myself and being able to to do that. That important activity of just sticking up for yourself, because at the end of the day, I often say to myself at work, if you don't look after yourself and think about yourself, nobody else necessarily will. They might, but you can't guarantee.

I

Did you, did you manage to? Did you find any? Like, I don't know. It's not easy. I realize it's, you know, the situation at work is always really busy and hectic. But did you ever, were you able to give yourself a hug at work? Did you, did you talk to yourself differently at work? You know, did you use the interventions themselves or practices at work, or did you just use, practice them at home?

R
Only only really at home, I just didn't really have the time. It's more important to me when I'm at work to free myself up, as the inpatient coordinator, the Doctor's can call you whenever and stress, giving myself poor, or practicing poor time management by taking, sort of, selfish time for myself, although it's essential, that would then, that could, sorry that could then stress me out at later point by making me busier when I could have got the handover down or something. So I would do that. But I have to say, [researcher] that that honestly you going through things and the knowledge of things, just just the knowledge of things and and being able to do it at home really honestly made made me feel better about the whole, about generally from day to day. I didn't have to necessarily practice it to reach upon that part of me.

I
Yeah, that's good. I just, I just haven't, an example. I just, I just remember when I was practicing some things and they were like you gotta do it every day and do it when you. And I just remember sitting on the toilet at work and going well, this is the only time I've got to do any of these things. And I was like, oh, it's not ideal, is it?

R

It's not.

I
It's just a, but it sometimes it is a real, you know, it can help to respond in the in the moment to those feelings to try and be self-compassionate or mindful or whichever, kind of, component, you know, whatever. But I was it was mindfulness that I was doing, so that's why. But um, it's a, yeah, it's it's not easy. Uh, so yeah, you've told, I think barriers and challenges, we've talked about a little bit in terms of using those um activities at work and positive experiences at work. I think you've covered all of those. So, unless you wanna talk about anything else, about work specifically, like about how that, anything to could be translated to work.

R
Um, I’d, I I guess I just want to say that I was, I was pleasantly surprised. That really has been my take home message, I think. That I I I do think, I didn't even have to give myself permission as much because when you have given yourself permission in the first instance and seen the benefits or experienced the benefits, then it makes more sense for you, um and it seems reasonable and so. So then yeah, so, so, so then you can, you can do that. That's that's my take home message I think.

I

OK. That's great. That's really helpful. Thank you. And did you think, did you, in you, in a way we've kind of gone backwards with my questions. Uh, we should have started with this one, at the… it.. have. Tell me, have you? What you've noticed about your attitude towards your work since this? Since this learning about self compassion, your attitude towards work. Are you able to talk about that at all, if that’s changed? Stayed the same or no different, not noticed.

R

Umm. I guess, thinking about it. It sounds funny and almost a bit backwards, but I I made the choice to help one of the HCA's cause the patient had a bed bath the other day. I didn't have to, but there there was somebody else doing it, but I wanted to do a full skin check, so it was, it was a good opportunity for me. So. Um. Your talk and and my knowledge of things did probably make me feel, make me feel better Umm, towards being able. I think when, I think when you have patients that are elderly or whatever and they've got more world wisdom, wordly wisdom about things, you think, hang on a minute, let let's look after them. It sounds like, I can't even properly articulate this but but knowing how knowing about the importance of self care made me think, Hang on, it's important too for my patients. And I know that I I know it's different that I'm not doing their self- care. I'm doing care because I can't do their self care. I'm just trying to practice um, caring for them because I can't do anything better than that, if that if it makes sense, I don't know.

I
Yeah, no that’s, I think it. I, I suppose what you're saying is that it's the compassion component that you were using with them? That you've learnt to use towards yourself is that fair? Is that, is that right? Kind of what you're trying to say, do you think?

R

Yeah, I I I I do think I think you you you you get me. Yeah. I I do think um, it it does help, you know, it, I think it does help with the patients and, well you hope, I mean this patient was severely demented a lot, dementia and things, I don't know if it was vascular dementia or what it was really or whether he’s had a stroke or whatever but, because I I just literally did this bed bath for them but um, I did think that um, it it can make us better nurses too. Just that knowledge. Yeah.

I
Yeah, yeah, I think it translates, doesn't it? If we know how it makes us feel when we're like that to ourselves, we also want to make other people feel like that, the way it makes us feel, so it helps to kind of, that makes sense. That makes a lot of sense, OK. Brilliant. Last question, I think then how do you feel that the training motivated you to share that teaching with others, if at all?

R
Well, as I said to you, the student with that learning outcome in in their, in their package, it was a S* student I believe. It was perfect because I I could. I could really talk her through it and actually it was quite fresh, um after when we initially did it, self-care. So it was quite fresh in mind. So then being able to talk to her about it, um, we didn't have enough time to fully talk about it, um, but definitely was, I definitely had some time and it was definitely very helpful and it made the student feel more cared for and and she even she even wrote to me and said I I felt like you really did care for me and care about my learning, not care for her as such, but care about her learning, her education and progression and consideration towards her learning and things. And I I do think that she she she's only like she she was young. I think she's only about 20 or 19 or something. She's quite young. And so she wasn't fully aware about this at all. She probably didn't even understand the the learning outcome in the book. So that that was definitely helpful.

I
Good, OK. Um. What? Let me read this. What kind of advice might you have for future participants in a training program? So if, like if I was giving it to one of your friends, the same session and you spoke to them beforehand, is there any advice that you might share with them about it or talk to, how would you kind of describe that or, yeah, advice specifically but future future participants, what would you say to them?

R

I can't help thinking that actually I've benefited from it because I really became engrossed when you went through it with me. I really engrossed myself into it and I, and I don't know that I would have got the same from it if I hadn't engrossed myself. So just to concentrate and to really try to really try to entertain the idea and and inbed the thoughts in your head and um try to the best of your ability to really partake in in the initial session.

I

Yeah.

R
Because you can't make anybody, you can't make anybody else take part, right? So you can bring a horse to water but you can't make it drink. They have to do it themselves, I think.

I

Yeah. Yeah, that's good. That's good advice. It's right, just, kind of, almost, kind of, keep an open mind, but make sure your your mind is there, you know, that you're not closing your mind off to it, kind of that kind of idea, isn't it?

R

I don't know if that isn't really the best advice, but for me personally I felt that.

I

OK, that's good. So yeah, that that is the end of the interview. So thank you very much. And is there anything you want to ask me about how it will work for, you know, in terms of analysis or there's anything that you want to ask about any of it particularly?

R

I would say it'd be interesting to know what you’re your You you best you your best advice as to what sort of resources or people or authors, whatever it may be, have you found that really, kind of, really seemed to be effective for you?

I

Yeah, I thought I, I don’t, yeah, I think we talked about Kristin Neff, didn't we, the, the and I think I put it. I'm I'm not. I felt like I needed to put it in a little, almost like a little take home sheet afterwards so that you could have looked at it. But Neff is NE double F.

R
Yeah, I've got Kristin Neff, but I just wondered if there is anything particular, any particular particular resource, any article or anything, anything to do, or any particular book that she's done or that anybody's done that you think is particularly good? Helpful.

I

Her website, her website I've turned to a few times when the self-compassion.org when it comes to thinking about trying different techniques for self compassion, I’d, sort of, start there. Loving kindness meditation is the one that I have, I went to first. If I'm honest, I connected mostly with that for self compassion and so loving kindness, we didn't have time to do to go through that and it and it does tend to be, a well it, it can, I don't know. I found it quite, the first time I did it, I found it quite difficult, right? Challenging emotionally. So I felt like maybe it's something we can kind of come to further down the line maybe. But loving kindness meditation is a kind of Buddhist meditation. Metta meditation. It's very kind of, very old school kind of Buddhist type meditation type and it specifically does bring out loving kindness, ur self compassion as a, sort of, outcome. So that, if you, I don't know if you've got a lot of access to meditations already, but if you're looking for a resource to to access a free meditations, you know, of various, an enormous number of meditations of different types and sleep meditations and self compassion exercise. I recommend Insight Timer as an app insight timer, and that's that's insight as in SIGHT.

R
Insight timer did you say?

I

Timer, yeah. Because I think I don't know why it's got that, it's a slightly strange name, but it I think because you can put in, oh, I've got 10 minutes. I'll just put a 10 minute timer on and it will bring up all the meditations that last for 10 minutes. And you can kind of go. OK, I've got 10 minutes or, you know. So I think that's maybe where the timer is. But so, but you can also put in the search term loving kindness and it will come up with all the loving kindness meditations or you can put, I tend to use it for sleep as well, so I'll put in sleep and it will, they, I I access my sleep meditation through that app as well so and I'm sure and Kristin Neff's has, does, I think she does podcasts on there as well. So you know it's kind of. There's lots and lots of resources through that app which I find, I I haven't even begun to really sort of get into it too deeply, but I I know that it's brilliant, so, well, from what I've tried. So yeah, those kind of things, if you're exploring these, these topics are good, good resources, definitely.

R

Thank you. That's really great. Really great. Thank you. Thanks.

I

That's good. I know it's a pleasure to share it because it's such a it's such a good, good thing, loving kindness meditation. If you're in it's it's it's really beautiful. So yeah, you've got my contact details. You've got my e-mail. And if there's anything you want to ask me, any other questions then really, honestly, feel free to get in touch anytime. That would be lovely. It doesn't always forward properly to my e-mail so I'm having to come into my uni emails but obviously Lucy’s there as well, so if you're struggling, you know, that's fine to get in touch with, you know, I'll get in touch with you or whatever. So yeah, please feel free.

R
Thank you. Thanks and thank you so much for this. And you're and your your time and everything it’s great. Thank you.

I
No, that's, it's, you know, I've really enjoyed it and it was really nice for me having you as my first person, way back, to do it because it, you know, you obviously it was the the kind of reception that you gave me was was amazingly appreciated, so thank you.

R

Yeah, Pleasure. You take care, good luck with your studies.

I

Thank you. You take care.

R

Take care then. Bye. Bye bye, [researcher] bye.
